# Family-Centered Care Improves Clinical Outcomes of Very-Low-Birth-Weight Infants: A Quasi-Experimental Study

**DOI:** 10.3389/fped.2019.00138

**Published:** 2019-04-12

**Authors:** Bo Lv, Xi-ronga Gao, Jing Sun, Tao-tao Li, Zhen-ye Liu, Li-hui Zhu, Jos M. Latour

**Affiliations:** ^1^Division of Neonatal Medicine, Hunan Children's Hospital, Changsha, China; ^2^Nursing School, Hunan University of Chinese Medicine, Changsha, China; ^3^Nursing Department, Hunan Children's Hospital, Changsha, China; ^4^Faculty of Health and Human Sciences, School of Nursing and Midwifery, University of Plymouth, Plymouth, United Kingdom

**Keywords:** neonatology, infants, very low birth weight, family-centered care, parents, patient outcomes

## Abstract

**Background:** Survival of very-low-birth-weight infants is improving in neonatology and family-centered-care might contribute to premature infants' clinical outcomes.

**Aim:** To evaluate a family-centered care intervention on clinical outcomes of very-low-birth-weight infants.

**Methods:** A quasi-experimental study was conducted in a Chinese NICU between June 2016 and June 2017. The intervention included parental education of basic care knowledge and skills followed by active participation in care for at least 4 h a day. A total of 319 very-low-birth-weight infants were recruited by convenience sampling; intervention group *n* = 156 and control group *n* = 163. Primary outcome measures were weight at discharge, length-of-stay, breastfeeding, nasal feeding, total parental nutrition, re-admission, hospital expenses. Secondary outcome measures were infant complications.

**Results:** Infants' weight at discharge was higher in the interventions group (2,654 g vs. 2,325 g, *p* < 0.001). Nutritional outcomes improved significantly: breastfeeding rate 139 vs. 91, *p* < 0.001; days of total parental nutrition 25 d vs. 32 d, *p* < 0.001; gastric feeding days 23 d vs. 35 d, *p* < 0.001. Length-of-stay and hospital expenses did not differ between both groups. Improved infants' complications were bronchopulmonary dysplasia (32 vs. 51, *p* = 0.031), retinopathy of prematurity (between groups no/mild and moderate/severe, *p* = 0.003), necrotizing enterocolitis (6 vs. 18, *p* = 0.019), and re-admission rate (21 vs. 38, *p* = 0.023). No differences were observed in intraventricular hemorrhage and nosocomial infections.

**Conclusion:** Very-low-birth-weight premature infants might experience improved clinical health outcomes when parents are present and caring from them. Family-centered care is as a beneficial care model for premature infants and should be recognized and implemented by NICUs where parents have currently limited access.

## Introduction

It is estimated that 15 million preterm neonates are born every year worldwide (1). With the implementation of the two-child policy in China in 2015, more preterm neonates are expected to be born (2). Preterm birth complications are the most frequent causes of deaths in children under 5 years of age (3, 4). Preterm infants born with very low birth weight (VLBW) experience several disadvantages and developmental challenges (5–7). Long-term growth development is one of these challenges. A 20-year follow-up study identified that males born with VLBW were significantly shorter and lighter as their female counterpart at 20 years of age (8). Although advances in medical technology has increased survival rates in neonatology, morbidity remains high and imposes emotional and financial burdens on families, society and healthcare system.

Family-centered care (FCC) is a care model in Neonatal Intensive Care Units (NICU) that has been implemented mainly in developed countries (9–11). The Institute of Patient- and Family Centered Care defines FCC as an approach to the planning, delivery, and evaluation of healthcare based on partnerships between health professionals, patients, and families (12). In pediatrics, FCC supports the involvement of parents in the care of their infant in the NICU (13). In NICU settings, the Newborn Individualized Developmental Care and Assessment Program (NIDCAP) is another approach to involve parents in the care (14). Evidence suggests that parents feel more empowered to care for their infant and improves short-term neurodevelopment of preterm infants (15). However, more studies are needed to prove the benefit of NIDCAP on long-term neurodevelopment (16).

Recent studies have demonstrated that providing parents training about the basic care of their infant improves infants' clinical outcomes (17, 18) Allowing parents to be involved in caregiving and becoming the primary caregiver might improve weight gain and breastfeeding rates (19). Despite the growing evidence of the benefit of FCC practices in NICU, it is not yet widely implemented in NICUs across the world (20–22).

In China, FCC in Chinese NICUs are at a developmental stage. In many Chinese NICUs parents are restricted to visit their infant or to participate in caregiving (23). However, recent studies from China reveal a growing trend in FCC practices and report clinical benefits to preterm infant (24–26). To date, there are limited reports presenting evidence of FCC practices in VLBW infants. Therefore, the aim of our study was to implement and test an FCC intervention providing parental education and participation in care among parents with VLBW infants. The hypothesis was defined as: parent education and parent participation in care improve clinical outcomes of VLBW infants.

## Materials and Methods

### Design

This study adopted a quasi-experimental design using convenience sampling. Due to the nature of delivering the intervention by nurses to parents and data collection, blinding was not possible. The study was conducted between June 2016 and June 2017.

### Setting

This study was conducted at the stand-alone Hunan Children's Hospital, Changsha, China. The Neonatology department includes five NICUs: Two level 3 tertiary care NICUs for term infants (45–60 beds) and preterm infants (45–60 beds) and three level 2 NICUs each 70 beds. The study was performed at the level 3 NICU for preterm infants with an annual admission rate of 600 infants. At time of the study our level 3 NICU had 60 beds operational due to high demands. The NICU is designed in three open space units with 20 incubators per unit. The NICU has one room with two incubators in case isolation for infection prevention is needed. Outside the NICU is a parent waiting room. Parents of VLBW infants are only allowed to visit their infant via video camera three times a week. In recent years, the NICU implemented the FCC caring model allowing only parents of stable preterm infants to visit the NICU and participate in the care. Based on the promising results of two studies among preterm infants (20, 21), the NICU decided to further implement FCC with parents of VLBW infants.

### Participants and Recruitment

Inclusion criteria were: preterm infants with a birth weight <1,500 g; non-invasive oxygen support; parents willing to participate in the care for at least 4 h a day. Exclusion criteria were: Infants with life-threatening congenital anomalies; surgery; palliative care; expected discharge within 1 week.

Infants and parents were recruited and assigned upon admission. At the start of the study, the first infant was recruited and assigned to the intervention group, the second infant to the control group and further recruitment took place in subsequent order.

### Intervention and Standard Care

The FCC intervention started by teaching parents the theoretical knowledge of basic care, infant development, hand hygiene, feeding methods, skin-to-skin contact, infection control. More specifically, basic care was instructed to involvement parents in bathing the infant, changing diapers, temperature measurement, and other basic care. Hand hygiene was instructed including the steps of handwashing and the times when to wash their hands. Breastfeeding was taught and promoted to parents. Skin-to-skin contact was taught to parents including kangaroo care and at the same time to communicate with the infant to promote infant-parent bonding. The teaching sessions lasted around 90 min and ongoing support to parents was provided throughout the admission period. After the maternal skills were assessed by the nurses, parents participated in the care for at least 4 h a day between 10.00 and 16.00 h. Nurses were trained from April to May 2016 to deliver the teaching sessions.

The standard care group followed the routine caring model and adhered to the hospital regulations. The routine standard care for parents of VLBW infants was restricted by the visiting policies; parents were not allowed to visit their infant and thus not involved in the care of their infant. Parents could come to the hospital 3 days a week; Monday, Wednesday, and Friday. During these visits, the neonatologist would meet the parents in a special room outside the NICU. During these meetings, an update of the health status of their infant was provided and parents were able to see their infant by video connection.

### Data Collection

Data were extracted from the infant's hospital records. Basic demographics of parents (mode of delivery, education and income levels) were collected. The primary outcome measures were: weight at discharge, NICU length-of-stay, breastfeeding rate, days of nasal feeding, days of total parental nutrition, re-admission within 1 month, hospital expenses. The secondary outcome measures were the infant complications: nosocomial infection rate, Bronchopulmonary dysplasia (BPD), Retinopathy of prematurity (ROP), Necrotizing enterocolitis (NEC), Intraventricular hemorrhage (IVH).

### Data Analysis

Data analysis was performed with IBM Corp. Released 2012. IBM SPSS Statistics for Windows, Version 21.0. Armonk, NY: IBM Corp. The Kolmogorov-Smirnow test was used to determine normal distribution of data. For descriptive statistics, mean, standard deviation percentages were applied. The Student *t*-test was used for continuous variable and the chi-square test for categorical variable. We defined statistical significance as *p* < 0.05. Data are presented as FCC group vs. Standard Care (SC) group.

### Ethics Considerations

This study was carried out in accordance with the recommendations of improving the reporting quality of nonrandomized evaluations of behavioral and public health interventions: the TREND statement, the Trend Group (27). The protocol was approved by the Ethics Committee of Hunan Children's Hospital (HCHLL-2015-33).

Parents were assured that their decision to withdraw or refuse to participate would not impact the care and treatment of their infant. All parents in the FCC group gave written informed consent in accordance with the Declaration of Helsinki. The infants and parents in the SC group received the routine care and parents were verbally informed and written informed consent were waived by the Ethics Committee.

## Results

### Characteristics of Infants and Parents

Of the 524 VLBW infants admitted to the NICU during the 12-month recruitment period. 319 met the inclusion criteria and were assigned to the FCC group (*n* = 156) or standard care group (*n* = 163). Both groups represented equal gender (male: 101, 64.76% vs. 102, 62.6%). The mean birth weight was not different between both groups (1,164 g, SD 211 vs. 1,204 g, SD = 196, *p* = 0.085). Gestational age of infants was significantly less the FCC group (28.9 weeks, SD = 1.6 vs. 29.4 weeks, SD = 2.3, *p* = 0.013). Apgar scores at 1 min and antenatal corticosteroid therapy did not reveal any statistical differences between both groups.

The characteristics of the parents did not show statistical differences in the mode of delivery (spontaneous vs. sectio-caesarea) and education levels. However, parents in the FCC groups had a higher monthly income than those in the SC group (<2,000 RMB, 11 vs. 26; 2,000–5,000 RMB, 69 vs. 81; >5,000 RMB, 76 vs. 56, *p* = 0.007).

### Infants' Clinical Outcomes

Discharge weight of infants in the FCC group was significantly higher than in the SC group. Similar statistical differences were observed with the NICU length-of-stay resulting in a shorter stay in the FCC group (Table 1). Three nutritional support outcome measures significantly improved in the FCC group; breastfeeding rate was higher, and the days of total parental nutrition and nasal feeding were lower in the FCC group (Table 1). The hospital expenses were lower in the FCC group, but not significant.

**Table 1:** Infants' Clinical outcomes.

**Outcomes**	**FCC group *n* = 156**	**SC group *n* = 163**	**Mean difference (95% CI)**	***P*-value**
Discharge weight (*g*; mean; SD)	2653.9 (672.2)	2324.9 (911.9)	329.0 (147.9–510.2)	<0.001
Length-of-stay (days; mean; SD)	60.6 (32.1)	63.2 (25.4)	−2.3 (−8.7 to 4.0)	0.474
Breastfeeding rate (*n*; %)	139 (89.1)	91 (55.8)	6.5 (3.6–11.7)	<0.001
TPN (days; mean; SD)	24.94 (19.5)	32.23 (19.9)	−7.3 (−11.4 to −3.2)	<0.001
Nasal feeding (days; mean; SD)	22.8 (16.4)	34.6 (27.2)	−11.8 (−16.9 to −6.7)	<0.001
Expenses (RMB; mean; SD)	83068 (36233.2)	88595 (18433.2)	−5527.0 (−20618.6 to 9564.7)	0.472

*FCC, Family-Centered Care; SC, Standard Care; TPN, total parenteral nutrition; Length-of-Stay, NICU Length-of-Stay; RMB, RenMinBi (Chinese Yuan)*.

### Infants' Complications

Table 2 presents the outcomes of the infant complication variables. Nosocomial infection and IVH rates did not present significant differences. In the FCC group were significantly less infants (*n* = 32) developing BPD compared to the SC group (*n* = 51). The FCC group had also less infants with no or mild ROP, less NEC and re-admissions compared to the SC group.

**Table 2:** Infants' complications.

**Complications**	**FCC group *n* = 156**	**SC group *n* = 163**	**Mean difference (95% CI)**	***p*-value**
Nosocomial Infection (*n*, %)	24 (15.3)	19 (11.6)	1.4 (0.7–2.6)	0.413
BPD (*n*, %)	32 (20.5)	51 (31.3)	0.6 (0.3–0.9)	0.031
**ROP (*****n*****, %)**				
No-ROP to mild ROP	135 (86.5)	119 (73.0)	reference	0.003
Moderate to severe ROP	21 (13.5)	44 (27.0)	2.4 (1.3–4.2)	
NEC (*n*, %)	6 (3.8)	18 (11.0)	0.3 (0.1–0.8)	0.019
Re-admission within 1 month (*n*, %)	21 (13.4)	38 (23.3)	0.5 (0.3–0.9)	0.023
**IVH (*****n*****, %)**				
No IVH, grade 1	146 (93.6)	147 (90.2)	reference	0.310
Grade 2-3	10 (6.4)	16 (9.8)	1.6(0.7–3.6)	

*FCC, Family-Centered Care; SC, Standard Care; BPD, Bronchopulmonary dysplasia; ROP, Retinopathy of prematurity; NEC, Necrotizing enterocolitis; IVH, Intraventricular hemorrhage*.

## Discussion

In the past decades, family-centered care has been implemented and further refined in NICUs in developed countries (22, 28, 29). In the Chinese context, barriers to implement FCC in NICUs have been vocalized as not enough space around incubators and lack of trained staff (30). However, FCC is gaining momentum among Chinese NICU clinicians. Our study is one of the few Chinese studies evaluating FCC in the NICU. We were able to successfully train nurses providing education and support to parents of VLBW infants. The infants' clinical outcome measures were promising in terms of infants' weight and nutritional support while complication rates of BPD, ROP and NEC have been less in the FCC group.

Several randomized controlled trials (RCTs) have been testing FCC interventions with parent educational programs in the NICU (22). Our results show some similarities with these studies. The recently published multi-center, multi-national FICare trial documented that weight gain of premature infants between the intervention and standard care groups increased faster and remained higher in the first 21 days of the trial (19). Although we did not include daily weight gain in our study, the VLBW infants in the FCC group had significantly higher weight at discharge while length-of-stay did not differ between both groups. However, three RCTs from China testing an educational FCC intervention confirmed also improved daily weight gain (31–33).

There is growing evidence that parental presence and involvement in care improves breastfeeding practices and nutritional outcomes of premature infants. Our study demonstrated an improvement in breastfeeding practices and less total parental nutrition and gastric feeding was necessary. A similar study confirmed our findings documenting that breastfeeding rates were higher in FCC group; 80.4 vs. 66.7%, *p* = 0.007 (17). A study in Taiwan showed that feeding practices improved in the FCC group; infants in the FCC group were significantly younger when they achieved full enteral feeding (95% CI = −1.9 to −0.2 weeks, *p* = 0.02) (18). Therefore, it can be assumed that parents can positively influence the feeding practices when providing them with sufficient educational and clinical support.

Allowing parents to be active partners in care might safeguard the preterm infants from complications. We demonstrated that VLBW infants in the FCC group had significantly less BPD, ROP, and NEC. The Stockholm Neonatal Family Centered Care Study, providing a new NICU environment for parents with individual rooms, reported that infants in the FCC unit had less moderate-to-severe BPD compared to the standard care unit; 3 vs. 11% (34). Rooming-in can be considered as a valuable contribution to FCC and it is noticed that many countries are transforming their NICUs into single-bedded rooms to allow rooming-in. Limited studies have explored the benefit of rooming-in in NICUs. A study looking at the impact of individual rooms demonstrated no significant short-term effect on parental stress and depression (35). However, another study documented a significant reduction in readmission rates (36). The clinical significance of rooming-in and single bedded designed NICUs cannot be underestimated anymore. With the results of our study and the vision to provide optimal care to infants and parents, our hospital is currently redesigning the NICU into more single-bedded FCC rooms.

The incidence of NEC in our study decreased from 18 to 6 in the FCC group. We have not tested the association of NEC and the nutritional variables. However, a meta-analysis of the effect of standardized feeding protocols and NEC, including nine studies with 4,755 infants, provide strong evidence to recommend early parental involvement in feeding practices (37). Additionally, providing parents with information might also affect their ability to care for their infant after discharge. The re-admission rate 1 month after discharge decreased significantly in our study. This is consistent in two other similar studies investigating an educational FCC intervention (25, 26).

Our study has several limitations. The sample size calculation and use of convenience sampling are limitations to address. Sample size was not calculated because we determined to run the data collection over a 12-month period using convenience sampling. The critique on this sampling strategy is that the study participants are not chosen at random which limits the generalizability of our results. Blinding of the intervention was not possible for parents and NICU staff due to the nature of the intervention where nurses provided education to parents and were on the unit all times. We did not collect any parent outcome measure which could strengthen the possible benefit of our intervention.

In conclusion, an FCC intervention, providing education to parents and empowering them to be involved in the daily care might improve clinical outcomes of VLBW infants. Despite the quasi-experimental design of our study, FCC is a beneficial care model for premature infants and should be recognized and implemented by NICUs where parents have limited access.

## Ethics Statement

This study was carried out in accordance with the recommendations of improving the reporting quality of nonrandomized evaluations of behavioral and public health interventions: the TREND statement, the Trend Group (23). The protocol was approved by the Ethics Committee of Hunan Children's Hospital (HCHLL-2015-33). Parents were assured that their decision to withdraw or refuse to participate would not impact the care and treatment of their infant. All parents in the FCC group gave written informed consent in accordance with the Declaration of Helsinki. The infants and parents in the SC group received the routine care and parents were verbally informed and written informed consent were waived by the Ethics Committee.

## Author Contributions

BL, LZ, XG, TL, and JML contributed to the design of the study, data collection, data analysis, and interpretation. JS and JML drafted the first manuscript. BL, ZL, and XG provided revisions. All authors contributed to manuscript revision, read and approved the submitted version.

### Conflict of Interest Statement

The authors declare that the research was conducted in the absence of any commercial or financial relationships that could be construed as a potential conflict of interest.
